# Regulation of Cu-Zn superoxide dismutase on SCN2A in SH-SY5Y cells as a potential therapy for temporal lobe epilepsy

**DOI:** 10.3892/mmr.2013.1790

**Published:** 2013-11-11

**Authors:** JUN XIANG, YUGANG JIANG

**Affiliations:** Department of Neurosurgery, Second Xiangya Hospital, Central South University, Changsha, Hunan 410011, P.R. China

**Keywords:** epilepsy, SCN2A, Cu-Zn superoxide dismutase, cortex, patch clamp

## Abstract

In order to evaluate SCN2A as a candidate gene for epileptic susceptibility and the use of a Cu-Zn superoxide dismutase (SOD) supplement as a potential therapy for epilepsy, SCN2A expression in the cortex and the correlation between SCN2A and Cu-Zn SOD in SH-SY5Y cells were examined. SCN2A expression and the concentration of Cu-Zn SOD in the cerebral cortexes of patients with primary and secondary temporal lobe epilepsy and normal brain cortex tissues were detected. By transfecting SH-SY5Y cells, the expression of SCN2A and the concentration of Cu-Zn SOD was analyzed and the single-cell patch clamp technique was employed in order to investigate the changes in sodium ion levels following SCN2A knockdown. SCN2A level restoration was also investigated with a Cu-Zn SOD supplement using an expression study and evaluated the changes in sodium ion levels following SCN2A knockdown. SCN2A expression and Cu-Zn SOD concentration decreased in the epileptic cerebral cortex. Following SCN2A knockdown, the concentration of Cu-Zn SOD declined and the si-SCN2A vector group showed a repeated discharge. Furthermore, the Cu-Zn SOD concentration was capable of restoring the expression of SCN2A following SCN2A knockdown in SH-SY5Y cells and the overexpression of Cu-Zn SOD prevented the repeated discharge caused by si-SCN2A. The results indicated that there is a low expression of SCN2A and Cu-Zn SOD in the epileptic cerebral cortex and provided novel insights into potential therapies for temporal lobe epilepsy.

## Introduction

Epilepsy is a common episodic neurological condition. In the majority of epilepsy patients, the predominant cause of the disorder is considered to be genetic rather than having an extraneous cause (1,2). The majority of epilepsy phenotypes are the product of interactions between genetic and environmental factors. A recent study focused on genetic variations that may affect the aetiology, prognosis and effects of epilepsy, such as the correlation between the condition and gene polymorphisms (1). To date, studies have shown that genetic mutations are correlated with epilepsy, particularly mutations of the sodium channel (1,3,4).

Voltage-gated sodium channels are crucial for membrane excitability. Mutations in the genes that code for the channel components are regarded to be key factors for epilepsy phenotypes. Voltage-gated sodium ion channels consist of two types of subunits, α and β subunits. Each α subunit is associated with one or more β subunit in order to form functional voltage-gated ion channels. These voltage-gated sodium channels are expressed in neurons and glia throughout the central and peripheral nervous system and have been highly conserved during the evolution of invertebrates and vertebrates (5,6). A SCN2A mutation has been identified to be associated with seizures, ataxia and a sensitivity to pain in epilepsy patients and patients with other neurological disorders (7). In mice, a mutation in the sodium channel SCN2A gene resulted in seizures and repetitive behaviors due to a persistent current (8). Altered sodium channel transcript levels in epilepsy patients reveal a potential role for sodium channels in the pathophysiology of epilepsy (9). Although a mutation in the sodium channel component has been demonstrated to induce epilepsy, the effect of altered SCN2A expression in epilepsy requires clarification.

In animals, oxygen radicals are produced as the byproducts of a normal oxidative metabolism (10). Therefore, in activated cells, more oxygen radicals were produced. Furthermore, neurons and glia are produced and reactive oxygen species are released (11). It has long been regarded that a controlled intracellular redox environment is crucial for proper cellular function. In order for cells to protect themselves from constant oxidative toxicity, cells have developed a defense system that ensures a balance between pro- and antioxidant molecules (12). Cu-Zn superoxide dismutase (Cu-Zn SOD) is a key enzyme in the dismutation of superoxide radicals, which result from the cellular oxidative metabolism process of cells, converting them into hydrogen peroxide (13). Increasing evidence suggests a role for oxidative stress in the manifestation of epilepsy (14–17). Sudha *et al*(14) reported that epileptic patients exhibited a low blood antioxidant status compared with the controls, suggesting that free radicals may be implicated in epilepsy. Ercegovac *et al*(18) speculated that the post-translational modification of existing functional proteins, particularly alterations to ion channels, may be partially responsible for the acute early changes in neuronal networks. However, the evidence that ion channels regulate oxidative stress action in patients with epilepsy remains limited.

In this study, the levels of SCN2A gene expression and the concentration of Cu-Zn SOD were investigated in the cerebral cortexes of patients with primary and secondary temporal lobe epilepsy and in normal brain cortex tissue. Furthermore, the changes in the SCN2A gene and Cu-Zn SOD concentration were analyzed following transfection with the si-SCN2A vector. In addition, co-transfection with the si-SCN2A vector and the overexpressed Cu-Zn SOD vector was performed in order to illustrate the effect of Cu-Zn SOD vector therapy on temporal lobe epilepsy.

## Materials and methods

### Subjects

In total, 20 patients with temporal lobe epilepsy (age, 35.12±10.69 years; 10 with primary and 10 with secondary) were recruited from the outpatient clinics of the Second Xiangya Hospital, Central South University (Changsha, China). A further 20 healthy controls (age, 40.59±9.77 years) were recruited from the medical staff of the Second Xiangya Hospital. This study was approved by the human ethics committee of the Central South University Xiangya Medical School (Changsha, China). Written informed consent was obtained from all patients/patients' families.

### Quantitative polymerase chain reaction (qPCR)

In this study the correlation between SCN2A mRNA and the temporal lobe was analyzed. All primers and probes were designed by Applied Biosystems (Carlsbad, CA, USA), and were hybridized between exons in order to avoid the amplification of genomic DNA. Total RNA isolation was performed using RNA TRIzol according to the manufacturer's instructions (Invitrogen Life Technologies, Carlsbad, CA, USA). By employing the Takara cDNA Library Construction kit, 1 μg total RNA was used to synthesize cDNA according to the manufacturer's instructions (Takara Bio, Inc., Shiga, Japan). The transcription levels for SCN2A and β-actin (a housekeeping gene) were quantified by the ABI 7500 realtime PCR system (Applied Biosystems). The amplification conditions were as follows: 95°C for 10 min, followed 40 cycles of 15 sec at 95°C and 1 min at 60°C, using TaqMan^®^ Universal PCR Master mix (Applied Biosystems). All results were normalized to the levels of β-actin RNA (TaqMan probes, Applied Biosystems). The relative expression level was calculated using the 2^−ΔΔCt^ method.

### Western blot analysis

Samples were separated in 10% SDS-PAGE gels and transferred to a polyvinylidene fluoride (PVDF) membrane (Millipore, Billerica, MA, USA). After blocking with 4% non-fat milk, SCN2A was detected by incubation with monoclonal anti-SCN2A antibody (SAB5200074, Sigma-Aldrich, St. Louis, MO, USA). β-actin was detected by the monoclonal anti β-actin antibody (A1978, Sigma-Aldrich). The anti-mouse IgG secondary antibody was conjugated to horseradish peroxidase and an enhanced chemiluminescent substrate (ECL plus, Amersham Pharmacia Biotech, Piscataway, NJ, USA) for signal development. The image was captured using a Fuji Film FLA 5000 image reader (Fuji Film, Stamford, CT, USA).

### Cu-Zn SOD detection

Tissues and cells were collected for enzyme concentration assays. Prior to performing the assays, the samples were thawed on ice and homogenized in PBS (Polytron PT3100; Kinematica AG, Littan, Switzerland). Cu-Zn SOD activities were assayed using a Cu/Zn Superoxide Dismutase ELISA kit (S2147, Sigma-Aldrich*)* according to the manufacturer's instructions.

### Single-cell patch clamp technique

The single-cell patch clamp technique was performed using the procedure as reported previously by Fertig *et al*(19). Briefly, before recordings, cells were transferred to a BX51WI (Olympus, Tokyo, Japan) upright microscope (equipped with infrared video microscopy) and incubated in DMEM/F12, 0.075% BSA, Pen/Strep/antimycotic, and 14 mM HEPES, pH 7.4. Individual cells were selected for recordings based on small round or ovoid cell bodies (diameter, 5–10 μm) and typically two or more extended processes. For each test, five patients with primary temporal lobe epilepsy and five with secondary temporal lobe epilepsy were involved. The methods used for cell culturing were also used to record the concentration of electrophysiological intracellular Ca^2+^. Cells were differentiated by exposure to the retinoic acid analogue, 4-[(5,6,7,8-tetrahydro-5,5,8,8-tetramethyl-2-napthalenyl), for six days. Whole cell patch clamp recordings were performed at room temperature from single cells, which were continually superperfused (2–3 ml/min) with phosphate-buffered saline (PBS). Ca^2+^ channel currents were elicited by stepping the membrane potential from a holding potential of −90 mV to +10 mV for 45 msec every 15 sec. Cells were continually perfused with a solution containing: NaCl 140 mM, KCl 2 mM, CaCl_2_ 2.5 mM, MgCl_2_ 1 mM, HEPES 10 mM, glucose 10 mM, sucrose 40 mM and bovine serum albumin 0.05%.

### Vector design and transient transfection

In order to compare the biophysical properties following the knockdown of SCN2A, the SCN2A interference vector was constructed following the protocol of a study by Tahiliani *et al*(20). To investigate the effect of Cu-Zn SOD on SH-SY5Y cells following transfection with a si-SCN2A vector, the overexpressed Cu-Zn SOD vector was constructed following the method provided by Hu *et al*(21).

SH-SY5Y cells, grown under standard culture conditions (5% CO_2_, 37°C) in Dulbecco's modified Eagle's medium supplemented with 10% fetal bovine serum, were transiently transfected with plasmids using Lipofectamine 2000 (Invitrogen Life Technologies) according to the manufacturer's instructions. Following 24 h of transfection, the cells were harvested and used in the following experiments.

### Immunofluorescence

SH-SY5Y cells were grown on 24 × 24 mm cover glasses and subsequently fixed in 4% paraformaldehyde solution in phosphate buffer for 30 min prior to 30 min incubation with a blocking reagent [5% fetal bovine serum in PBS]. The primary antibodies of SCN2A (anti-SCN2A, SAB5200074; Sigma-Aldrich) were incubated overnight at 4°C prior to being washed with PBS. Immunofluorescence staining was performed with secondary antibodies conjugated to fluorescein isothiocyanate (F5262, Sigma-Aldrich). A conventional fluorescence microscope (Axiovert 100; Carl Zeiss, Oberkochen, Germany) was used for visualization.

### Effects of SCN2A interference on SH-SY5Y cells

To investigate the biophysical properties of SH-SY5Y cells following SCN2A knockdown, the cells were planted into 24-well plates. The wells were divided into three groups (8 wells/group): The control group, the empty vector control group and the si-SCN2A vector group. Following transfection, SCN2A expression was detected by qPCR, western blotting and immunofluorescence. Cu-Zn SOD concentrations were assayed. Furthermore, single-cell patch clamp techniques were used to analyze the electrophysiological changes of cells in the three groups.

### Effect of Cu-Zn SOD on SCN2A knockdown in SH-SY5Y cells

The effect of Cu-Zn SOD on SH-SY5Y cells following transfection with a si-SCN2A vector. The wells were divided into four groups (6 wells/group), including the control group, the empty vector control group, the si-SCN2A vector group and the si-SCN2A vector + Cu-Zn SOD vector group. Following 24 h of transfection, SCN2A expression was analyzed by qPCR, western blotting and immunofluorescence. Cu-Zn SOD concentrations and cell electrophysiological changes were also assayed using the single-cell patch clamp techniques.

### Statistical analysis

All values are provided as the mean ± SD. Continuous variables that did not have a Gaussian distribution underwent log transformation. Student's t-test was used to compare the differences between groups. One-way analysis of variance was used to determine the differences among the groups. P≤0.05 was considered to indicate a statistically significant difference. If F-ratios exceeded the critical value (P≤0.05), Newman-Keul's post hoc test was performed in order to compare the groups.

## Results

### Lower levels of SCN2A expression and low Cu-Zn SOD content in the cortex of patients with temporal lobe epilepsy

**Figure 1:**
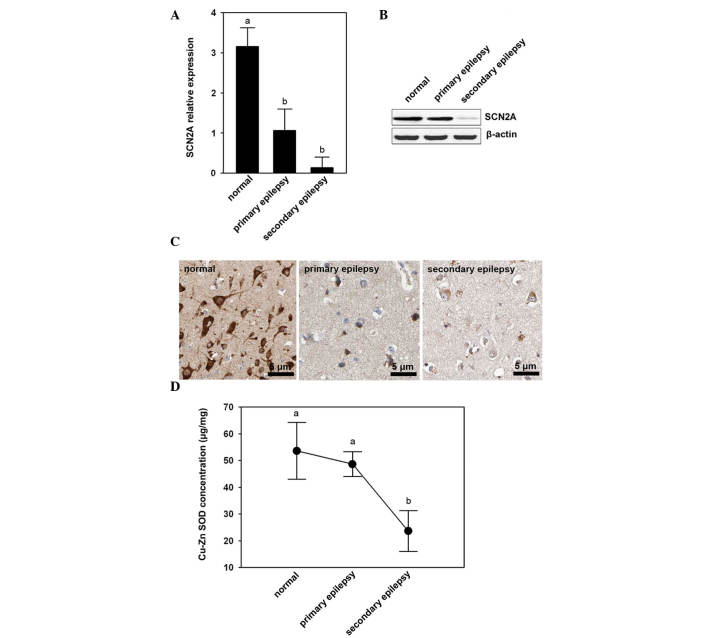
Lower levels of expression of SCN2A and Cu-Zn superoxide dismutase (SOD) in patients with secondary epilepsy. (A) Significantly lower expression levels of SCN2A mRNA in patients with primary and secondary epilepsy was observed by quantitative polymerase chain reaction. Different characters showed significant difference among the groups (P<0.05). (B) Lower protein expression in patients with secondary epilepsy by western blotting. (C) Significant signals were observed in normal tissues compared with the cerebral cortexes of patients with primary and secondary temporal lobe epilepsy by immunohistochemistry. (D) Cu-Zn SOD concentration among the normal cortex tissues, the cerebral coretex tissues of patients with primary temporal lobe epilepsy and those with secondary temporal lobe epilepsy. Different letters (a or b) show significant differences among the groups (P<0.05).

The differential expression of SCN2A was assessed in the cerebral cortex of patients with primary and secondary temporal lobe epilepsy and normal brain cortex tissue. Using qPCR, it was confirmed that of the three different types of tissues, the normal brain cortex tissue demonstrated a markedly higher expression of SCN2A transcripts (P<0.05), while the cerebral cortex of patients with primary and secondary temporal lobe epilepsy exhibited lower expression of SCN2A transcripts (Fig. 1A). Furthermore, the cerebral cortex of patients with secondary temporal lobe epilepsy had weaker western blotting reactions compared with normal brain cortex tissue and cerebral cortex of patients with primary temporal lobe epilepsy (Fig. 1B). Similar to the studies of mRNA and protein expression levels, immunofluorescence revealed that the cerebral cortex of patients with primary and secondary temporal lobe epilepsy had weaker signals (Fig. 1C) when compared with normal brain cortex tissue.

### Cu-Zn SOD concentration in the three tissue types

The results showed that the cerebral cortexes in patients with secondary temporal lobe epilepsy had lower Cu-Zn SOD concentrations than the normal brain cortex tissue (P<0.05) and cerebral cortex of patients with primary temporal lobe epilepsy (Fig. 1D).”

### SCN2A knockdown suppresses Cu-Zn SOD

**Figure 2:**
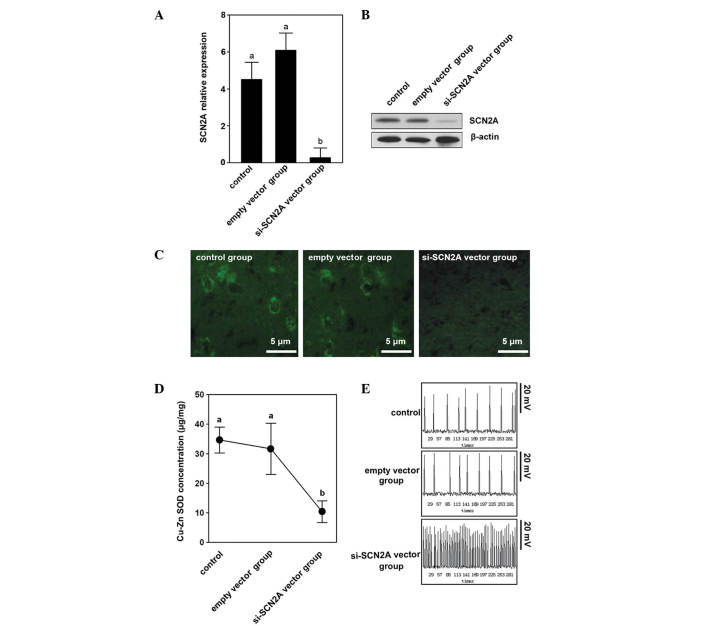
Effects of si-SCN2A vector transfection on Cu-Zn superoxide dismutase (SOD) expression and resting membrane electric potential. (A and B) Downregulated transcript and protein expression levels of of SCN2A following transfection with the si-SCN2A vector, respectively. (C) No significant signals were observed in the si-SCN2A vector groups by immunofluorescence. (D) Cu-Zn SOD concentrations among the control, empty vector and si-SCN2A vector groups. Different letters (a or b) show significant differences among the groups (P<0.05). (E) Resting membrane electric potential detected by patch clamp showed a repeated discharge caused by si-SCN2A.

In order to elucidate the regulation of SCN2A by Cu-Zn SOD, the SCN2A interference vector was used to transfect SH-SY5Y cells and detected the concentration of Cu-Zn SOD in the different groups. SCN2A expression was determined by qPCR, western blotting and immunofluorescence. The control and empty vector control groups demonstrated higher mRNA and protein expression levels compared with the si-SCN2A vector group (Fig. 2A and B). The immunofluorescence result indicated that the si-SCN2A vector group had weaker signals compared with the control and the empty vector control groups (Fig. 2C). A notable difference was identified in the si-SCN2A vector group among the three groups that were studied.

The Cu-Zn SOD concentration in the si-SCN2A vector group was 11.23±4.52 μg/mg, which was significantly lower than the control and empty vector control groups (P<0.05; Fig. 2D).

The control and empty vector control groups showed a stable resting membrane electric potential (53.34±3.25 mV and 52.68±3.48 mV) with a discharge frequency of approximately once every minute. However, the si-SCN2A vector group showed repeated discharge, which was similar to the phenomenon of epilepsy. The volatility (between 120–150 mV) and frequency (6–10 times/min) were regular (Fig. 2E).

### Poor efficacy of Cu-Zn SOD in SCN2A knockdown SH-SY5Y cells

**Figure 3:**
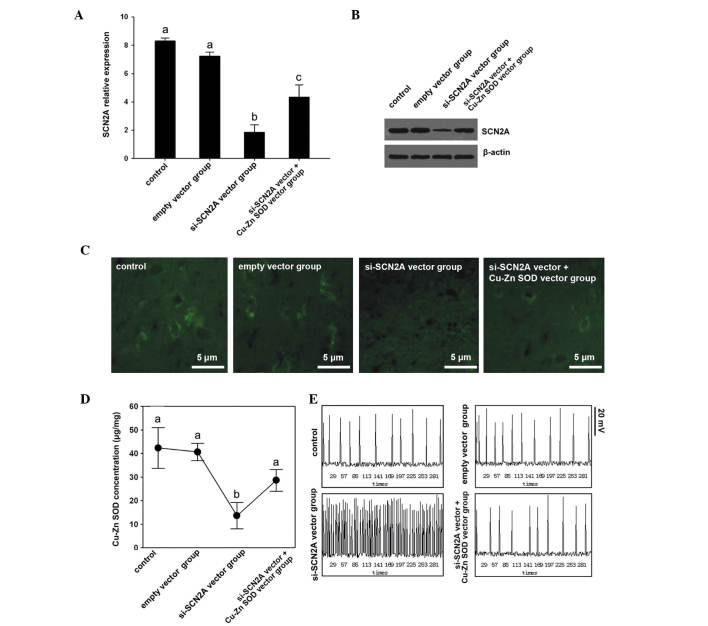
Effects of Cu-Zn superoxide dismutase (SOD) on si-SCN2A vector transfection induced resting membrane electric potential. (A and B) Downregulated transcript and protein expression levels in SCN2A following transfection with the si-SCN2A vector, respectively. (C) No significant signals were observed in the si-SCN2A vector groups by immunofluorescence compared with the other groups. (D) Cu-Zn SOD concentration among the control, empty vector and si-SCN2A vector groups. Different letters (a or b) show significant differences among the groups (P<0.05). (E) Resting membrane electric potential detected by single-cell patch clamp showed a repeated discharge caused by si-SCN2A while the overexpression of Cu-Zn SOD prevents the repeated discharge.

The response of SH-SY5Y cells following the knockdown of SCN2A was also evaluated in order to assess the efficacy of Cu-Zn SOD with a deficiency of SCN2A. Four groups were employed in this study, including the control, the empty vector control, the si-SCN2A vector and the si-SCN2A vector + Cu-Zn SOD vector groups. The expression levels of SCN2A were assayed in order to confirm the silencing efficiency. mRNA and protein levels showed a significant decrease following si-SCN2A vector transfection (Fig. 3A and B). Notably, SCN2A mRNA expression levels were significantly lower in the si-SCN2A vector group compared with the si-SCN2A vector + Cu-Zn SOD vector group (Fig. 3A and B). Furthermore, the distribution results of SCN2A (Fig. 3C) indicated that the si-SCN2A vector group and the si-SCN2A vector + Cu-Zn SOD vector group had weaker signals compared with the other two groups.

### Cu-Zn SOD concentrations of the groups

The si-SCN2A vector group exhibited the lowest concentration of Cu-Zn SOD compared with the si-SCN2A vector + Cu-Zn SOD vector group, the control group and empty vector group (Fig. 3D). The empty vector control group had the highest concentration of Cu-Zn SOD while the lowest concentration of Cu-Zn SOD was observed in the si-SCN2A vector group (Fig. 3D).

The resting membrane electric potential of the si-SCN2A vector group was similar to that identified in a previous study (8). Notably, the overexpression of Cu-Zn SOD rescued the repeated discharge caused by si-SCN2A, which demonstrated that Cu-Zn SOD is beneficial for the low expression of SCN2A in SH-SY5Y cells (Fig. 3E).

## Discussion

The correlation between voltage-gated sodium channels and epilepsy has been widely reported (22). The Scn2aQ54 transgenic line may be capable of inducing sodium-channel-dependent epilepsy in a mouse model (8). In this study, Scn2aQ54 heterozygotes exhibited adult-onset, progressive epilepsy that begins with partial seizures of short duration of hippocampal origin. The causal mutation, GAL879–881QQQ, in the cytoplasmic S4–S5 linker of domain 2 results in delayed channel inactivation and increased persistent current. Thus, the genome mutation has been revealed. However, the SCN2A expression changes in epilepsy have not been discussed, particularly with reference to the cerebral cortexes of patients with primary or secondary temporal lobe epilepsy and normal brain cortex tissues. In the present study, in terms of RNA levels and protein levels, normal brain cortex tissues exhibited a higher expression of SCN2A than the cerebral cortexes of patients with primary or secondary temporal lobe epilepsy. This result suggested that in epileptic tissues, there is a lower level of expression of SCN2A. In organisms, voltage-gated calcium channels allow for the conversion of electrical signals to chemical signals, which, due to the input of calcium ions (Ca^2+^) then induce the release of neurotransmitters from synaptic vesicles (23). SCN2A encodes the α-subunit of the voltage-gated sodium channel (24). Thus, the lower levels of SCN2A expression may contribute to disorder in the transmission of electrical signals to chemical signals in the cortex, which induces pathological changes. Similar results were observed in a study of the Cu-Zn SOD concentration that was higher in epileptic tissues than normal brain cortex tissue (25). Cu-Zn SOD has been regarded as the first-line of defense against free radical damage (26). Abnormal concentrations of Cu-Zn SOD may induce neurotoxicity, which results in exacerbations (27). Subsequently, the Cu-Zn SOD concentrations were investigated following SCN2A knockdown in SH-SY5Y cells in order to understand the correlation between Cu-Zn SOD and SCN2A.

Following SCN2A knockdown with transfection of the si-SCN2A vector, SCN2A expression decreased significantly. These results demonstrated that the SCN2A knockdown was efficient and successful. Furthermore, the result also demonstrated that the concentration of Cu-Zn SOD was downregulated following a reduction in the levels of SCN2A. To date, little information has been provided regarding the effect of SCN2A expression on Cu-Zn SOD in organisms or cells. Therefore, the present study provided a novel insight in to Cu-Zn SOD and its regulation by SCN2A in epilepsy; the decrease in SCN2A expression may reduce the concentration of Cu-Zn SOD in the brain cortex, which contributes to serious trauma to the cortex. The resting membrane electric potential was also analyzed by single-cell patch clamp. The cells, which had been transfected with the si-SCN2A vector, exhibited a repeated discharge, similar to that identified in epilepsy. The result suggested that the low expression of SCN2A in the cerebral cortexes of patients with primary and secondary temporal lobe epilepsy induced the repeated discharge. Thus, the upregulation of SCN2A expression in the cortex may be an effective treatment for the repeated discharge that occurs in epilepsy.

It has been reported that Cu-Zn SOD is crucial in preventing oxidative damage to cells (28). Thus, a decrease in Cu-Zn SOD levels in the cortex induces damage and a Cu-Zn SOD supplement may repair the damage caused. In addition, it was observed that in cortex tissue of epileptics, the SCN2A expression was abnormal, with a lower expression than that in normal cortex tissue. Subsequently, the protective effect of Cu-Zn SOD on SH-SY5Y cells was analyzed following SCN2A knockdown. Notably, the Cu-Zn SOD supplement group demonstrated a higher level of SCN2A expression compared with the group that received no treatment, which indicated that the addition of Cu-Zn SOD may be capable of reducing the depression induced by SCN2A knockdown. As demonstrated, the low expression of SCN2A induced repeated discharge. Subsequently, the effect of Cu-Zn SOD overexpression on si-SCN2A-induced repeated discharge was observed. Notably, the overexpression of Cu-Zn SOD prevented the repeated discharge due to si-SCN2A. Thus, it was presumed that Cu-Zn SOD may be capable of treating the repeated discharge, which may be a feasible treatment strategy for epilepsy.

In the present study, it was confirmed that SCN2A expression was lower in epileptic cerebral cortexes. This change may induce pathological changes in the cortex tissue. In SH-SY5Y cells, SCN2A expression was downregulated artificially by knockdown technology. With the low expression of SCN2A in the cell lines the concentration of Cu-Zn SOD also decreased. No information has been reported regarding the regulation of Cu-Zn SOD in the cerebral cortex by SCN2A thus far. SCN2A variation in the genome or expression levels leads to tissue injury, due to the abnormal release of neurotransmitters from synaptic vesicles. Thus, in the cerebral cortex, tissue injury is caused by SCN2A disorder and the downregulation of Cu-Zn SOD concentration. However, whether SCN2A may be capable of regulating Cu-Zn SOD directly requires further study. Conversely, Cu-Zn SOD was capable of rescuing SCN2A in SCN2A knockdown cell lines. This result indicates that a Cu-Zn SOD supplement may be an effective therapy for epilepsy but the mechanism underlying this requires further study. Furthermore, it was demonstrated that Cu-Zn SOD may prevent the repeated discharge that is caused by the low expression of SCN2A.

In conclusion, a significant decline in the expression of SCN2A and the concentration of Cu-Zn SOD was observed in the cerebral cortex of epileptic patients and the decrease in SCN2A expression may be restored by increasing the Cu-Zn SOD. However, further investigation and a larger cohort are required to investigate the underlying molecular mechanism.
